# Dominance of Emerging G9 and G12 Genotypes and Polymorphism of VP7 and VP4 of Rotaviruses from Bhutanese Children with Severe Diarrhea Prior to the Introduction of Vaccine

**DOI:** 10.1371/journal.pone.0110795

**Published:** 2014-10-20

**Authors:** Sonam Wangchuk, Marcelo T. Mitui, Kinlay Tshering, Takaaki Yahiro, Purushotam Bandhari, Sangay Zangmo, Tshering Dorji, Karchung Tshering, Takashi Matsumoto, Akira Nishizono, Kamruddin Ahmed

**Affiliations:** 1 Public Health Laboratory, Department of Public Health, Ministry of Health, Thimphu, Bhutan; 2 Department of Microbiology, Faculty of Medicine, Oita University, Yufu, Japan; 3 Department of Pediatrics, Jigme Dorji Wangchuk National Referral Hospital, Thimphu, Bhutan; 4 Department of Pediatrics, Mongar Regional Referral Hospital, Mongar, Bhutan; 5 Research Promotion Institute, Oita University, Yufu, Japan; Universidad Nacional de La Plata., Argentina

## Abstract

A prospective study was performed to determine the molecular characteristics of rotaviruses circulating among children aged <5 years in Bhutan. Stool samples were collected from February 2010 through January 2011 from children who attended two tertiary care hospitals in the capital Thimphu and the eastern regional headquarters, Mongar. The samples positive for rotavirus was mainly comprised genotype G1, followed by G12 and G9. The VP7 and VP4 genes of all genotypes clustered mainly with those of neighboring countries, thereby indicating that they shared common ancestral strains. The VP7 gene of Bhutanese G1 strains belonged to lineage 1c, which differed from the lineages of vaccine strains. Mutations were also identified in the VP7 gene of G1 strains, which may be responsible for neutralization escape strains. Furthermore, we found that lineage 4 of P[8] genotype differed antigenically from the vaccine strains, and mutations were identified in Bhutanese strains of lineage 3. The distribution of rotavirus genotypes varies among years, therefore further research is required to determine the distribution of rotavirus strain genotypes in Bhutan.

## Introduction

Bhutan is a small landlocked country located between India and China. Part of the population is concentrated in the capital Thimphu, but most are scattered sparsely throughout the country. Diarrhea is a major cause of illness and death in Bhutanese children, and the morbidity rate from diarrhea in <5 years old children is 314.6/1,000 population [1], [2], while 13% of deaths are attributable to diarrhea [3]. Approximately 83% of the population have access to safe drinking water and 91% have access to sanitary toilets [1]. A shift from bacterial to possibly viral diarrhea has been observed, although information regarding childhood diarrhea is scarce.

Rotavirus is a major cause of childhood diarrhea throughout the world, and is responsible for 114 million infections and 453,000 child deaths per year [4]. Rotavirus has also been associated with in nondiarrheal diseases [5]. Two rotavirus vaccines are available at present, RotaTeq (Merk & Co. Inc., Whitehouse Station, NJ, USA) contains G1–4 and P[8] antigens, while the other Rotarix (GlaxoSmithKline Biologicals, Brussels, Belgium) contains G1 and P[8] antigens, and both have been used successfully in several countries [6]. RotaTeq is a live, attenuated vaccine composes of five bovine-human reassortant strains [7]. The monovalent live attenuated Rotarix vaccine is based on the human rotavirus strain RIX 4414 [7]. Worldwide, genotype G1 is dominant, followed by G2–4 and G9, and these genotypes are mainly responsible for rotavirus-related diarrhea [8]. In Bhutan, the genotype distribution is unknown, which has hindered the policy discussion on introduction of rotavirus vaccine by policy makers. Thus, understanding the distribution patterns of circulating genotypes and their relationships with rotaviruses in neighboring countries might facilitate effective rotavirus control in Bhutan by introducing vaccination. Given the level and distribution of the available health services in Bhutan, the introduction of rotavirus vaccine might help to reduce child morbidity, and mortality. However, in contrast to other countries, it would be very challenging for Bhutan to cope with the possibly undesirable outcomes that might be associated with rotavirus vaccines [9]–[17], and the environmental monitoring to determine the spread of vaccine-like strains. Thus, a more cautious approach to the introduction of rotavirus vaccine may be appropriate for Bhutan. A cautious approach does not mean that Bhutanese children will be excluded from rotavirus vaccination; it means that policy makers should be aware of the benefit and risk of vaccination. In the present study, we determined the antigenic characteristics of G1 strains, and we performed a phylogenetic analysis of the rotaviruses circulating in Bhutan.

## Materials and Methods

### Sample collection

This study was undertaken at Jigme Dorji Wangchuk National Referral Hospital (JDWNRH), Thimphu and Mongar Regional Referral Hospital (MRRH), Mongar. This is part of a project to identify the etiology of viral diarrhea in Bhutanese children [18], [19]. JDWNRH is the only national reference hospital in the country, and serves the population of Thimphu. MRRH is a regional referral center in the east region, and mainly serves the population of Mongar. Stool samples were collected prospectively from children <5 years attended at the outpatient and inpatient departments of these hospitals with watery diarrhea. A case of diarrhea was defined as three looser than normal stool during a 24 hr period. The samples obtained from JDWNRH were collected between February 2010 and January 2011, while those from Mongar were obtained on April 26, 2010.

### Detection of rotavirus, genotyping and electropherotyping

A commercial enzyme-linked immunosorbent assay (Rotaclone, Meridian Diagnostics, Cincinnati, OH, USA) was used to detect rotavirus antigen in stool samples. Genomic RNA was extracted from the rotavirus positive stool samples using a QIAamp Viral RNA Mini Kit (Qiagen, Hilden, Germany) to determine the G and P types [20]. RNA was extracted with phenol-chloroform-isoamyl alcohol and used to determine the electropherotypes by polyacrylamide gel electrophoresis (PAGE) [21], [22].

### Nucleotide sequencing and phylogenetic analysis

The nucleotide sequences of the VP7 and VP8* portions of VP4 genes were determined using a BigDye Terminator v3.1 Cycle Sequencing Kit (Applied Biosystems, Gaithersburg, MD, USA). The purified amplicons were sequenced using an ABI3130 Genetic Analyzer (Applied Biosystems). All of the procedures were conducted according to the manufacturer’s instructions.

A multiple sequence alignment was performed using CLUSTALW ver. 2 [23]. Phylogenetic analyses were conducted with the neighbor-joining method using MEGA software ver. 5 [24]. The branching patterns were evaluated statistically based on bootstrap analyses of 1,000 replicates. Nucleotide sequences of different strains of rotavirus were obtained from the GenBank.

### Ethical statement

This study was approved by the Research Ethics Board of Health, Bhutan (www.health.gov.bt/rebh.php). Informed verbal consent was obtained from the guardians on behalf of the children enrolled in this study. The verbal consent was not recorded. Stool sample collection is a routine work for diarrheal cases and left over samples after routine investigation was used in the present study, besides it is not an invasive procedure, does not cause pain or harm during collection, and is not a life-threatening process therefore written consent was not obtained from the guardians. The ethics board approved the consent procedure.

## Results

**Figure 1:**
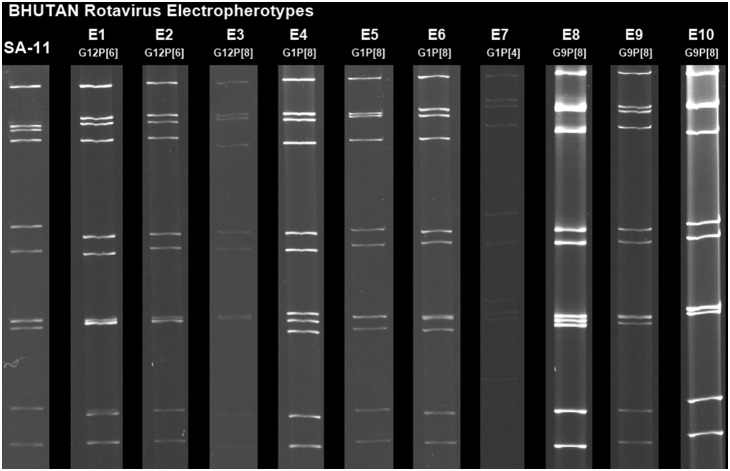
Electropherotypes of rotaviruses identified in Bhutan. On the extreme left, SA-11 indicates the electropherotype of strain Sa-11, which was used as a marker in each electrophoresis run. In total, 10 electropherotypes were identified, E1–E10. The genotype of each electropherotype is shown below the electropherotype. With the exception of E7, the electropherotypes had long patterns. Among five strains from Mongar, three were electropherotype E4, one E10, and one was untypable.

In total, 44/123 (35.8%) stool samples were positive for rotavirus, and ten electropherotypes, i.e., E1–E10, were detected in 38 samples (Figure 1). The frequencies of the G1, G9, and G12 genotypes were 26 (59.1%), five (13.4%), and 13(29.5%), respectively. The relative detection rates for different combinations of G and P genotypes were in the following order: G1P[8] = 21 (47.7%), G12P[6] = 10 (22.7%), G9P[8] = 5 (11.3%), G1P[4] = 5 (11.3%) and G12P[8] = 3 (6.8%).

**Figure 2:**
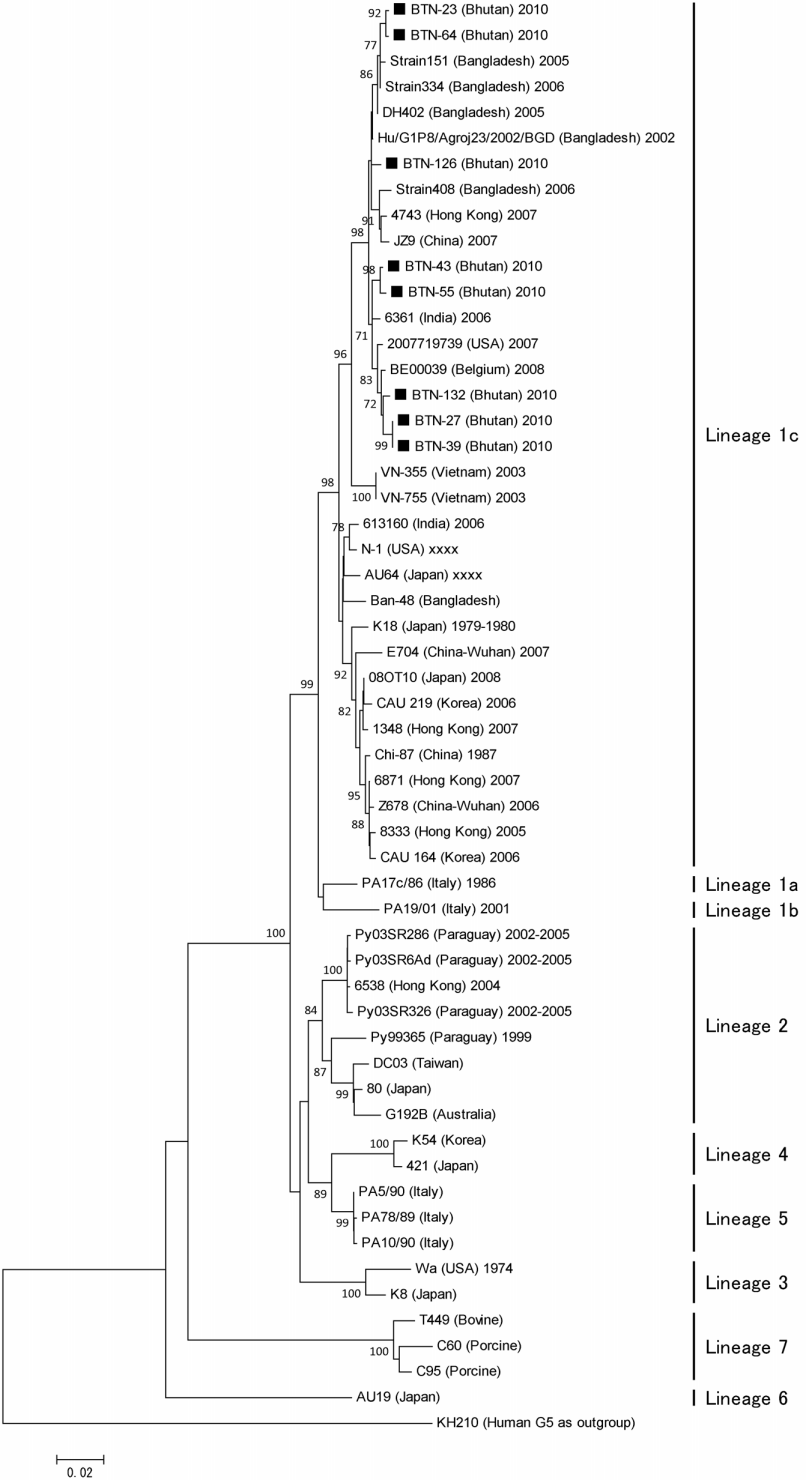
Phylogenetic tree constructed based on the deduced amino acid sequences of the VP7 genes of G1 strains. Bhutanese strains are indicated by black squares, which are followed by the strain numbers. Human rotavirus KH210 (G5) was used as an outgroup. The numbers adjacent to nodes represent the bootstrap values; values <70% have not shown. The scale bar shows the genetic distance, which is expressed as amino acid substitutions per site. The DNA Data Bank of Japan/European Molecular Biology Laboratory/GenBank accessions numbers are: AB905455 (rotavirus strain BTN-23), AB905456 (BTN-64), AB905457 (BTN-55), AB905458 (BTN-126), AB905459 (BTN-43), AB905460 (BTN-132), AB905461 (BTN-27), and AB905462 (BTN-39).

Of the eight lineages of G1 rotaviruses, the Bhutanese strains belonged to lineage 1c and were closely associated with strains from Bangladesh, India, Belgium, and the USA, thereby indicating that strains similar to Bhutanese G1 clones are also circulating both in the neighboring countries and in distant parts of the world (Figure 2). The phylogenetic analysis indicated that Bhutanese G1 possibly originated from a single clone or similar clones before changing via point mutations. The shared nucleotide and amino acid identities of the VP7 gene were 98–100% among Bhutanese G1 strains.

**Figure 3:**
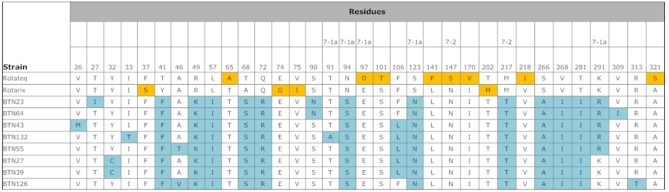
Comparison of the antigenic residues of VP7 present in genotype G1 strains of RotaTeq and Rotarix, and the strains circulating in Bhutan. The respective antigenic epitopes are shown above the residue numbers. The amino acid residues in the Bhutanese strains that differed from those in the vaccine strains are highlighted in blue. The amino acid residues highlighted in yellow indicate that the residue is different from the other vaccine strain and Bhutanese strains.

Compared with the VP7 sequences of Rotarix strain RIX4414 [16] and RotaTeq strain W179-9 [25], the Bhutanese G1 strains had 94 and 93% shared amino acid identities. Figure 3 shows the details of the amino acid substitutions. Compared with RotaTeq, there were amino acid substitutions in 19 residues of all Bhutanese strains, and 11 additional residues were also substituted in some Bhutanese strains. Five of these residues belonged to the 7-1a epitope of the antigenic region [26], where substitutions, in three residues can cause neutralization escape [16], and two of the residues belonged to the 7-2 antigenic region, which is also responsible for neutralization escape. Compared with Rotarix, amino acid substitutions were found in 15 residues of all Bhutanese strains, as well as additional substitutions in 11 other residues in some Bhutanese strains. Four of these residues were located in the 7-1a antigenic region, three of which are responsible for neutralization escape. Only one residue belonged to the 7-2 antigenic region, which is responsible for neutralization escape.

**Figure 4:**
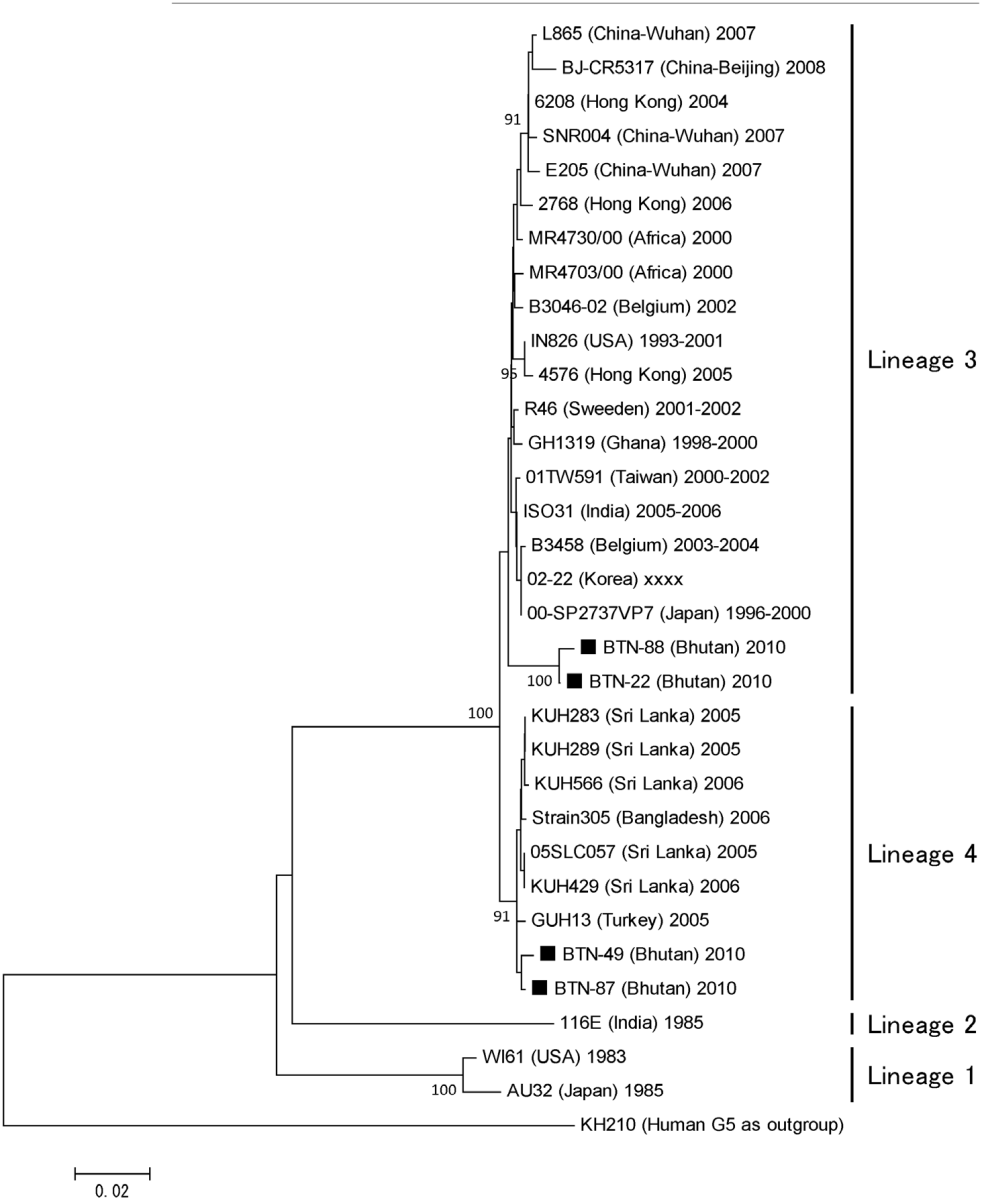
Phylogenetic tree constructed base on the deduced amino acid sequences of the VP7 genes of G9 strains. Bhutanese strains are indicated by black squares, which are followed by the strain numbers. Human rotavirus KH210 (G5) was used as an outgroup. The numbers adjacent to nodes represent the bootstrap values; values <70% are not shown. The scale bar shows the genetic distance, which is expressed as amino acid substitutions per site. The DNA Data Bank of Japan/European Molecular Biology Laboratory/GenBank accessions numbers are: AB905463 (rotavirus strain BTN-22), AB905464 (BTN-49), AB905465 (BTN-87), and AB905466 (BTN-88).

Bhutanese strains belonged to lineages 3 and 4 among the four lineages of G9 rotaviruses (Figure 4). The lineage 3 strains formed an independent cluster, which was closely association with a cluster of strains from Korea, Japan, Belgium, Ghana, India, Taiwan, and Sweden. The lineage 4 strains formed a cluster with strains from Sri Lanka, Turkey, and Bangladesh. The shared nucleotide and amino acid identities of the VP7 gene among the Bhutanese G9 strains were 96–99% and 97–100%, respectively.

**Figure 5:**
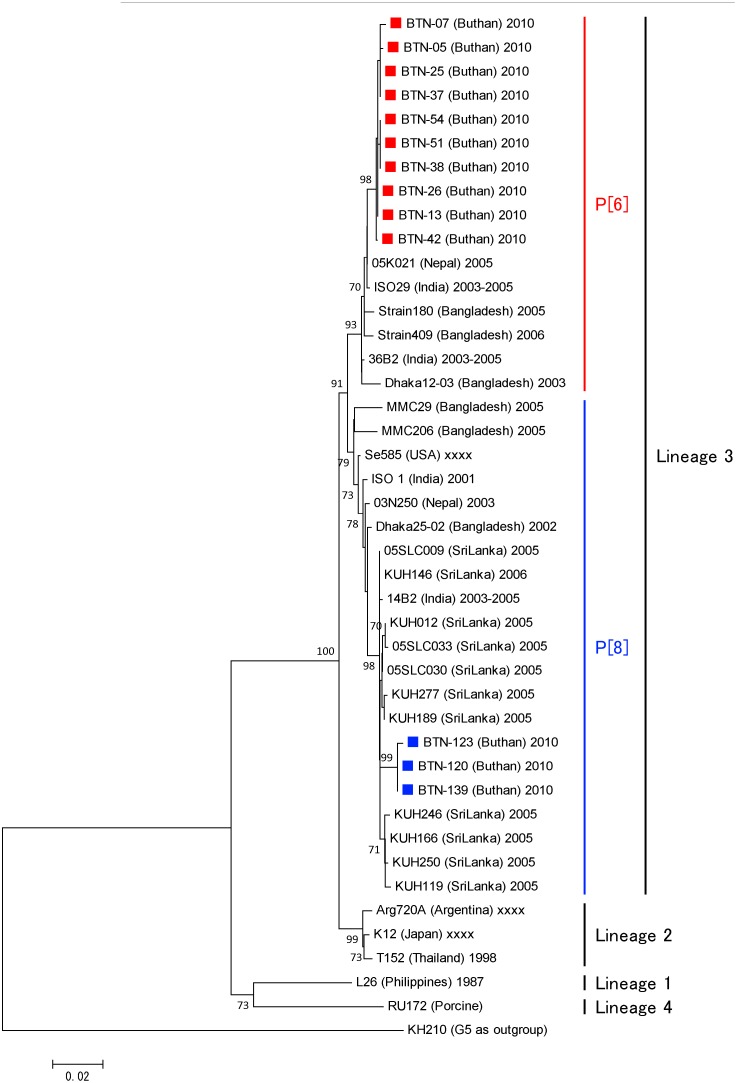
Phylogenetic tree constructed based on the deduced amino acid sequences of the VP7 gene of G12 strains. Bhutanese strains are indicated by black squares, which are followed by the strain numbers. Human rotavirus KH210 (G5) was used as an outgroup. The numbers adjacent to nodes represent the bootstrap values; values<70% are not shown. The scale bar shows the genetic distance, which is expressed as amino acid substitutions per site. The DNA Data Bank of Japan/European Molecular Biology Laboratory/GenBank accessions numbers are: AB905467 (rotavirus strain BTN-05), AB905468 (BTN-07), AB905469 (BTN-13), AB905470 (BTN-25), AB905471 (BTN-26), AB905472 (BTN-37), AB905473 (BTN-38), AB905474 (BTN-42), AB905475 (BTN-51), AB905476 (BTN-54), AB905477 (BTN-120), AB905478 (BTN-123), and AB905479 (BTN-139).

Phylogenetic analysis of the G12 strains showed that lineage 3 segregated into two clusters with a bootstrap value of 90%, where one cluster comprised G12P[8] strains and the other comprised G12P[6] strains (Figure 5). The Bhutanese G12P[8] strains clustered with Sri Lankan and Indian strains. The Bhutanese G12P[6] strains clustered with Sri Lankan, Indian, Nepalese, and Bangladeshi strains. The shared nucleotide and amino acid identities of the VP7 gene among the Bhutanese G12 strains were 96–100% and 97–100%, respectively. The shared nucleotide and amino acid identities of the VP7 gene were 99–100% among the Bhutanese G12 P[6] strains. The shared nucleotide and amino acid identities of the VP7 gene were 99–100% among the Bhutanese G12 P[8] strains.

**Figure 6:**
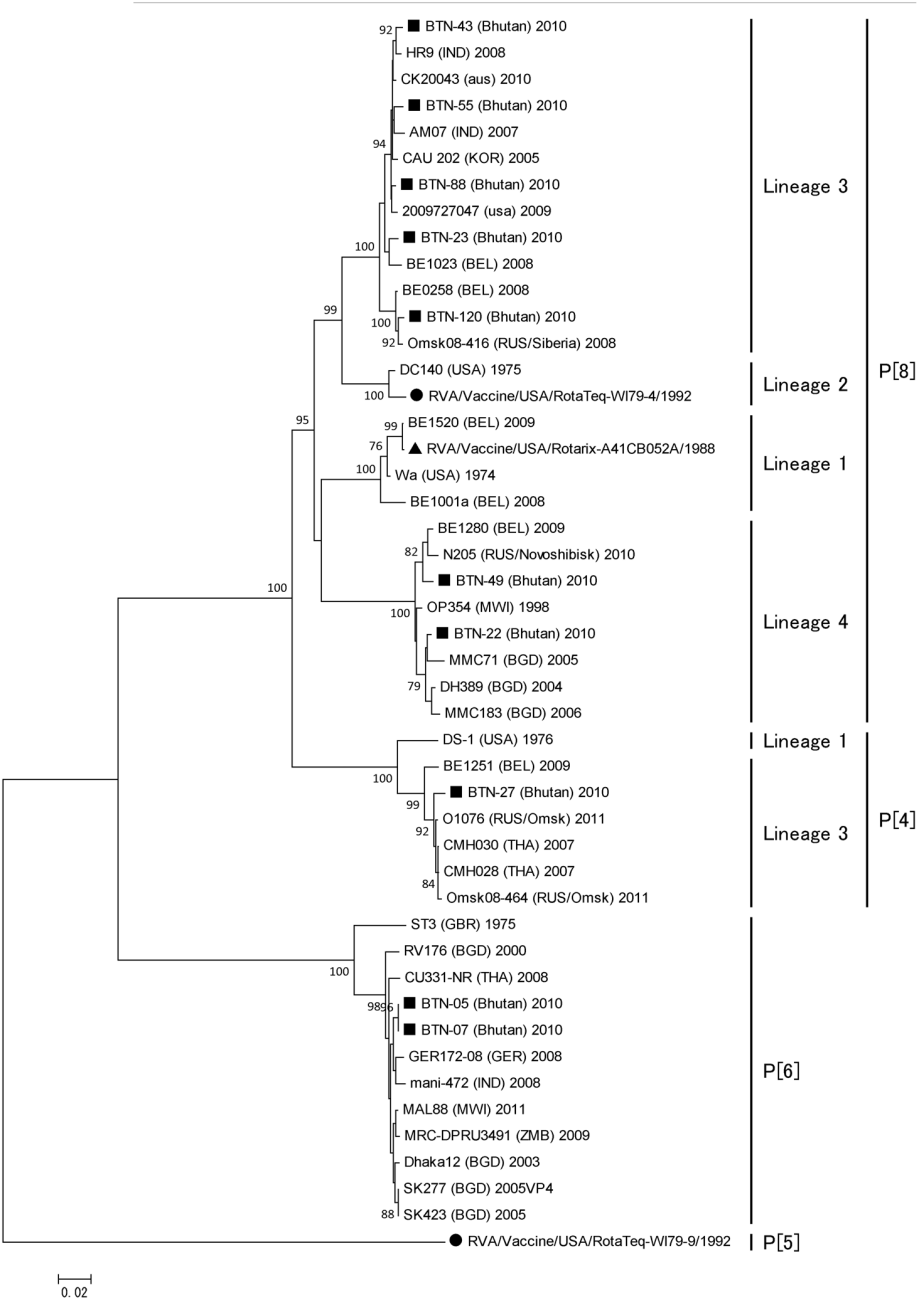
Phylogenetic tree constructed based on the deduced amino acid sequences of the VP8* genes of G1, G9, and G12 strains from Bhutan and global P[8], P[6], P[5], and P[4] strains. The species and country of origin are shown in parentheses after the strain name. The numbers adjacent to node represent the bootstrap values; values<70% are not shown. The scale bar shows the genetic distance, which is expressed as amino acid substitutions per site. The DNA Data Bank of Japan/European Molecular Biology Laboratory/GenBank accessions numbers are: AB905368 (rotavirus strain BTN-05), AB905369 (BTN-07), AB905370 (BTN-120), AB905371 (BTN-23), AB905372 (BTN-88), AB905373 (BTN-49), AB905374 (BTN-22), AB905375 (BTN-27), AB905376 (BTN-43), AB905377 (BTN-55).

**Figure 7:**
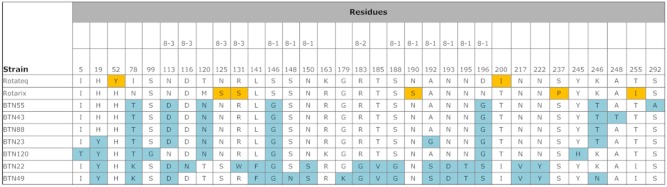
Comparison of the antigenic residues in the VP8* head of VP4 from RotaTeq and Rotarix, and strains circulating in Bhutan. The respective antigenic epitopes are shown above the residue numbers. The amino acid residues in Bhutanese strains that differed from those in the vaccine strains are highlighted. The amino acid residues highlighted in yellow indicate that the residue is different from the other vaccine strain and Bhutanese strains. BTN-22 and BTN-49 belong to lineage 4 of genotype P[8].

According to the phylogenetic analysis, P[8], P[6], and P[4] of the Bhutanese strains belonged to the globally circulating strains and were closely associated with strains from several different countries, but mainly with those from India and Bangladesh. Bhutanese P[8] belonged to lineages 3 and 4 (Figure 6). The VP8* head of VP4 contains four (8-1 to 8-4) surface-exposed antigenic epitopes, which have been predicted to comprise 25 amino acids [16], [27]. All of the Bhutanese P[8] contained G at residues 146, instead of S, which are found in the two vaccine strains. Compared with RotaTeq, there were amino acid substitutions in 6 residues of all Bhutanese strains, and 10 additional residues were also substituted in some Bhutanese strains. Compared with Rotarix, amino acid substitutions were found in 9 residues of all Bhutanese strains, as well as additional substitutions in 9 other residues in some Bhutanese strains. Eleven amino acids of Bhutanese P[8] that belonged to lineage 4 differed from the vaccine strains (Figure 7).

## Discussion

The proportion of rotavirus-positive samples detected in Bhutanese children is comparable to that found in neighboring India [28] and Bangladesh [21]. Given the rising cost of health care, the introduction of a rotavirus vaccine might reduce the burden on the health-care system. However, our study demonstrated the high diversity of rotavirus strains in Bhutan, where only the G1, G9, and G12 genotypes are in circulation, which might pose a challenge for the efficacy of rotavirus vaccines. Furthermore, the lower efficacy of rotavirus vaccine in the in low-income countries of Asia and Africa is another challenge [29], [30]. The exact cause of this lack of efficacy in low-income countries is largely unknown, but it has been suggested that it may be attributable to the diversity of rotavirus strains, the passage of maternal antibodies to babies via breast-feeding, the presence of other viral agents, and malnutrition [31], [32], all of which might be present in Bhutan.The VP7 sequences of Bhutanese G1 strains had low shared amino acid sequence identities with those of Rotarix RIX4414 and RotaTeq strain W179-9 and there were more amino acid differences compared with RotaTeq than with the Rotarix strain. With respect to the amino acid differences that are known to be responsible for generating neutralization escape strains, the Bhutanese strains contained five amino acid differences relative to RotaTeq and four relative to Rotarix. These suggest that Rotarix may be a better choice, although the mechanism responsible for vaccine-induced immunological protection is not clearly understood. Serum and intestinal serotype-specific neutralizing antibodies directed against VP7 and VP4, and virus-specific cytotoxic T lymphocyte induction are responsible for protection, although other proteins may be involved in immune protection against rotavirus [33], [34].

The VP4 of Bhutanese strains also differed compared with the vaccine strains. In Rotarix and RotaTeq, P[8] belongs to lineages 1 and 2, respectively, whereas those of the Bhutanese strains belonged to lineages 3 and 4. Lineage 4 of genotype P[8], which is also called OP354-like (P[8]b) VP4, was first detected in Malawi [35], and it has also been detected in India [36], Bangladesh [37], Thailand [38], Vietnam [39], and Finland [40]. Overall, Bhutanese P[8] exhibited divergence compared with the vaccine strains, although there were greater divergences among lineage 4 of the P[8] strains. These results highlight the need to extend this study to the determination of further VP4 nucleotide sequences in more strains from Bhutan to evaluate the differences among the antigenic epitopes.

No previous studies are available, so it is not known whether the dominance of G9 and G12 is attributable to natural fluctuations in rotavirus genotypes or if they represent a unique situation in Bhutan. The emergence of G9 and G12 in Bhutan indicates that no barriers are able to prevent the spread of emerging strains to any corners of the world. According to the phylogenetic analysis, the Bhutanese strains were most closely associated with Indian and Bangladeshi strains, thereby reflecting the close relationships between these countries in terms of commodities and travel. This close association was also found in the two main clusters of G12 strains, which comprised G12P[8] and G12P[6] strains, indicating that the G12 genotype strains circulating in this region are probably derived from two clones. The phylogenetic analysis also showed that Bhutanese P[6] was derived from human strains rather than porcine strains. In the present study, we did not detect any animal derived strains, although >70% of Bhutan is covered with forest and interactions with wild animals are common, particularly via shared water sources that may be contaminated by animal excreta. Thus, further research may be required to detect rotavirus infections caused by animal strains or reassortants.

The detection of 10 different electropherotypes among 38 electropherotyped samples in our study might represent substantial diversity of rotaviruses circulating in Bhutan as compared with Turkey (5/38) [41], Sri Lanka (18/74) [22], Hong Kong (35/432) [42], and Bangladesh (15/88) [21], respectively. The concentration of populations in urban areas in different parts of the country and their interactions may have generated this high diversity in Bhutan and helped these strains to spread successfully in children. The factors responsible for the high diversity of strains and the unusual genotype distributions found in Bhutan are complex. It is necessary to establish a nationwide surveillance system before introducing rotavirus vaccine into Bhutan. Furthermore, a vaccine trial may be required to evaluate the efficacy in Bhutanese children before selecting a specific vaccine. Both should be considered because of the complex vaccine-induced selection pressure on rotavirus strains.
